# Association Between E-Cigarette Use (Vaping) and Oral Health in Adolescents and Young Adults: A Systematic Review

**DOI:** 10.3390/jcm15103886

**Published:** 2026-05-18

**Authors:** Carmen Machuca-Portillo, Carolina Caleza-Jiménez, Cira Suárez-Marchena, Lucy Chandler-Gutiérrez, Pablo Relimpio-Pérez, María José Barra-Soto, Lydia López-del Valle, Juan J. Segura-Egea

**Affiliations:** 1 Department of Stomatology, Pediatric Dentistry Division, School of Dentistry, University of Sevilla, C/Avicena s/n, 41009 Sevilla, Spain; mmachuca@us.es (C.M.-P.); chandler@us.es (L.C.-G.); prelimpioperez@gmail.com (P.R.-P.); mbarra@us.es (M.J.B.-S.); 2 School of Dental Medicine, Building San Juan Medical Center, Medical Sciences of University of Puerto Rico, San Juan, PR 00936, USA; lydia.lopez1@upr.edu; 3 Department of Stomatology, Endodontic Division, School of Dentistry, University of Sevilla, C/Avicena s/n, 41009 Sevilla, Spain; segurajj@us.es

**Keywords:** electronic nicotine delivery systems, students, adolescent, oral health

## Abstract

**Background:** Vaping has become one of the most prevalent risk behaviors among adolescents and young adults worldwide. Although electronic cigarettes (ECs) are often perceived as safer than conventional tobacco, concerns regarding their impact on oral health are increasing. This systematic review aimed to critically evaluate and synthesize the available evidence regarding the association between e-cigarette use and oral health outcomes in adolescents and young adults. **Methods:** A systematic search of PubMed/MEDLINE, EMBASE, Scopus, and EBSCOhost databases was conducted for observational studies published within the last ten years. Studies evaluating oral health outcomes among adolescents and young adults were included. Risk of bias was assessed using the Joanna Briggs Institute Critical Appraisal Tool for Analytical Cross-Sectional Studies, and certainty of evidence was graded using the GRADE approach. **Results:** Eight observational studies met the inclusion criteria and were included in the qualitative synthesis. E-cigarette use was associated with increased caries risk, higher prevalence of gingival inflammation, alterations in salivary flow and pH, changes in oral microbiota—including increased levels of Porphyromonas gingivalis—and elevated frequencies of micronuclei in oral mucosal cells. Four studies were rated as having low risk of bias and four as moderate risk. The overall certainty of evidence was low for all outcomes due to methodological limitations, heterogeneity in outcome assessment, and inadequate control of confounding factors, including dual use of combustible tobacco products. **Conclusions:** Current evidence may suggest a possible association between e-cigarette use and adverse oral health alterations in adolescents and young adults. However, given the low certainty of evidence, residual confounding, and the predominance of cross-sectional designs, causal relationships cannot be established. Well-designed longitudinal studies that clearly differentiate exclusive e-cigarette users from dual users and adequately adjust for behavioral confounders are required to clarify the independent impact of vaping on oral health in this vulnerable population.

## 1. Introduction

The definition of a Nicotine Delivery Device (NDD), commonly known as an electronic cigarette (EC) or electronic nicotine delivery system (ENDS), refers to a product or any of its components that allows the consumption of vapor containing nicotine through a mouthpiece [1,2].

The use of this type of device is called vaping, as many people believe that it produces vapor which is then inhaled. However, ECs actually generate an aerosol of tiny particles, which differs from the conventional concept of vapour [3,4].

The term “vaper” emerged as a marketing strategy to avoid the use of “electronic cigarette,” associated with the stigma of traditional cigarettes, and to link the product to the perceived safety of water vapor. It is important to highlight the diversity of these products, which come in a wide range of shapes, colours, and flavours, potentially increasing their appeal to consumers [3,5].

Despite being marketed as safer alternatives to conventional tobacco, growing evidence suggests that e-cigarettes are not risk-free and may have adverse health effects [6]. Vaping has emerged as one of the most prevalent risk behaviours among adolescents worldwide [7]. Current research examines prevalence patterns, determinants of initiation, associated health effects, and the extent to which vaping serves as a gateway to other substance use. Global meta-analyses estimate that approximately 18–22% of students have ever used an EC or ENDS, and 8–10% report current use [1,5,7]. Prevalence is highest in high-income countries and among older adolescents and males. In several national surveys, past-30-day use among high school students has reached 20–30% in recent years [1,7,8,9]. Consistently identified predictors of vaping include older age, male sex, greater discretionary income, and peer use. Many adolescents perceive ECs—particularly flavoured, disposable, or highly marketed devices—as less harmful than conventional cigarettes [10]. Vaping is also strongly associated with other risk behaviours, including alcohol and cannabis use, tobacco experimentation, sexual risk behaviours, and delinquency [11,12,13].

Systematic reviews and umbrella reviews link adolescent vaping to respiratory symptoms, asthma exacerbation, cardiovascular alterations, and mental health concerns, although long-term evidence remains limited [14,15]. Notably, oral health outcomes have been comparatively understudied in adolescent vaping research. Most available studies employ cross-sectional designs and focus primarily on dental caries rather than periodontal health, limiting the ability to infer causal relationships [16]. Prospective studies and meta-analyses may indicate that non-smoking adolescents who vape have higher odds of subsequently initiating cigarette smoking and other substance use, suggesting potential downstream risks for periodontal disease, oral cancer, and additional oral pathologies. Patterns of use have intensified over time, with a substantial subset of adolescents progressing to frequent or habitual vaping [3,17,18,19,20].

Vaping exposes the oral cavity to heated aerosols containing nicotine, flavouring agents, aldehydes, and heavy metals. Although current evidence may suggest that ECs produce fewer oral health harms than conventional cigarettes, they are nonetheless associated with significantly greater adverse effects than those observed in non-users [21].

Recent increases in adolescent vaping have raised concerns regarding its potential effects on periodontal health. Emerging evidence from contemporary studies and systematic reviews indicates that, although conventional cigarette smoking remains the most significant behavioral risk factor for periodontal disease, EC use is also associated with unfavorable periodontal outcomes. Reported effects include heightened gingival inflammation, early clinical attachment loss, and alterations in the oral microbiome that may predispose youth to periodontal pathology. Nonetheless, the magnitude of these risks appears lower than that associated with traditional smoking, and some investigations may suggest that clinical manifestations in adolescents may be less pronounced or inconsistent. Current findings are constrained by methodological limitations—most notably the predominance of cross-sectional designs, reliance on self-reported measures, and the scarcity of longitudinal research in adolescent populations—highlighting the need for more robust evidence to clarify long-term impacts [22,23].

The principal oral health consequences associated with e-cigarette use include an increased risk of dental caries, as well as gingival and periodontal diseases. Evidence suggests that individuals who use e-cigarettes may exhibit higher levels of dental plaque, greater gingival inflammation, and measurable clinical attachment loss compared with non-smokers, although the extent of periodontal damage generally appears to be lower than that observed in conventional cigarette smokers. These alterations may be accompanied by changes in the oral microbiome that could predispose individuals, particularly younger populations, to periodontal pathology [3].

In addition, several systematic reviews suggest a potential increase in dental caries risk among e-cigarette users, possibly mediated by enhanced biofilm accumulation, the presence of sugary or viscous components in certain e-liquids, and shifts in the oral microbiome toward more cariogenic bacterial profiles. Beyond caries and periodontal involvement, commonly reported symptoms include oral and pharyngeal irritation, xerostomia, gingival discomfort, and mucosal alterations such as nicotinic stomatitis, hairy tongue, and hyperplastic candidiasis [24].

Despite the growing body of literature examining the systemic and respiratory effects of e-cigarette use, evidence regarding its impact on oral health in adolescents and young adults remains limited and fragmented. Most available studies have focused on adult populations or have evaluated isolated oral outcomes without comprehensive synthesis. Furthermore, adolescents represent a particularly vulnerable group due to ongoing biological development, high exposure to flavored products, and evolving patterns of nicotine dependence. To date, no systematic review has specifically evaluated and critically appraised the association between e-cigarette use and oral health outcomes in this population.

Previous studies have suggested that e-cigarette use may influence oral health by affecting salivary composition and the oral microbiota, potentially promoting dysbiosis and increasing susceptibility to oral disease [25].

Therefore, the aim of this systematic review was to assess and synthesize the available evidence from observational studies investigating the association between vaping and oral health alterations—including caries risk, periodontal parameters, salivary changes, and mucosal alterations—in adolescents and young adults. Young adults were defined as individuals aged up to 28 years, consistent with epidemiological classifications of transitional age groups between adolescence and adulthood.

## 2. Materials and Methods

### 2.1. Protocol and Registration

This systematic review was conducted in accordance with the Preferred Reporting Items for Systematic Reviews and Meta-Analyses (PRISMA) 2020 guidelines [20,26].

The review protocol was prospectively registered in the International Prospective Register of Systematic Reviews (PROSPERO) under the registration number CRD420261304162. The protocol is publicly accessible at [https://www.crd.york.ac.uk/PROSPERO/view/CRD420261304162] (accessed on 8 February 2026). No major deviations from the registered protocol were made during the conduct of this review.

### 2.2. Review Question

This systematic review aimed to conduct a systematic review based on PRISMA guidelines (Table S1) to assess the literature evidence based in the last ten years related to the impact of vaping or EC in oral health of adolescents. The primary research question of this systematic review was: “In adolescents and young adults, is exposure to e-cigarette use associated with increased risk of oral health problems compared with non-users?”.

This question was developed using the PECOS framework [27] as follows:

Population: Adolescents and young adults
Exposure: Exposure to e-cigarette use (vaping)
Comparators: No exposure to e-cigarette
Outcomes: Oral health problems (caries risk, periodontal diseases, mucosa alterations, gingivitis…).

Young adults were defined as individuals aged up to 28 years, in accordance with the eligibility criteria applied.

### 2.3. Eligibility Criteria

Eligibility criteria were defined a priori according to the PECOS framework. Studies meeting all the following inclusion criteria were considered eligible:

- Study design: observational studies as cross-sectional, case–control and prospective or retrospective cohort studies.
- Studies published within the last 10 years.
- Studies evaluating oral health problems in adolescents and young adults.
- Studies published in English.
- Studies including a minimum sample size of 50 participants.

Although case–control and cohort studies were considered eligible, only cross-sectional and retrospective observational studies met the inclusion criteria.

Exclusion criteria:

- Clinical trials, Systematic Reviews, Meta-analysis, Case reports, Case series, reviews, letters, editorials, and commentaries.
- Studies not aligned with the objectives of the present review.

### 2.4. Search Strategy

A comprehensive electronic search strategy was implemented to identify studies evaluating the association between e-cigarette use (vaping) and oral health outcomes in adolescents and young adults. The initial search was conducted in January 2026 and was updated through 3 February 2026. The databases searched included PubMed/MEDLINE, Scopus, Embase, and WoS.

To reduce the risk of publication bias, grey literature sources were also explored through Google Scholar and the OpenGrey repository. In addition, the reference lists of all included articles and relevant reviews were manually screened to identify further eligible studies.

The search strategy combined controlled vocabulary (e.g., MeSH terms) and free-text keywords and Medical Subject Headings (MeSH) terms: “Electronic Nicotine Delivery Systems”, “Students”, “Adolescent” and “Oral Health”related to electronic nicotine delivery systems, adolescents and young adults, and oral health outcomes. Boolean operators (“AND” and “OR”) and truncation were used to combine search terms, maximize sensitivity and ensure comprehensive retrieval of relevant studies. The search strategy was adapted for each database according to its specific syntax and interface requirements. The full database-specific search strategies, including all terms, operators, and applied limits, are provided in Table 1. The last search was conducted on 3 February 2026. Only studies published in English were included.

### 2.5. Selection of Studies

Articles were selected by three authors individually (C.C.-J., C.M.-P., and L.V.-L.). References of the included articles were also hand-searched. All three authors examined the title and abstract of the articles found to determine their eligibility and then analyzed the full text of those that appeared to meet the criteria to finally make the decision to include or exclude them. Disagreements about eligibility were resolved by discussion and consensus. Full texts of all selected studies were then obtained. A manual search of reference lists from the selected articles was also performed to identify additional relevant studies.

### 2.6. Data Extraction

Two of the authors (C.C.-J. and C.S.-M.) were responsible for data extraction, while three reviewers (C.C.-J., L.V.-L. and C.M.-P.) verified the tabulated data to ensure the absence of typo errors and carried out the analysis of the articles; the articles in disagreement were discussed. To analyze and synthesize the data, the following details were extracted from the studies: author, year and country of publication, sample size, age range, oral aspects studied, methods for studying oral status and results/conclusions and summarized on tables. When available, additional variables related to potential confounders were extracted, including smoking status (exclusive e-cigarette use versus dual use), oral hygiene behaviors, and other relevant exposure characteristics. However, these variables were not consistently reported across studies.

### 2.7. Data Synthesis

A meta-analysis was not performed due to substantial heterogeneity across the included studies. Specifically, there was considerable variability in study design (cross-sectional and retrospective), exposure definitions (lack of consistent differentiation between exclusive e-cigarette users and dual users), and outcome assessment methods, which included clinical examinations, self-reported measures, salivary parameters, microbiological analyses, and cytological findings. In addition, the included studies reported outcomes using different metrics and did not provide sufficiently comparable quantitative data (e.g., effect sizes, confidence intervals) to allow for statistical pooling. Pooling such heterogeneous data could have resulted in misleading or non-interpretable summary estimates.

Therefore, a narrative synthesis was undertaken. The findings were systematically organized according to predefined outcome domains, including clinical outcomes (e.g., caries and periodontal parameters), biological outcomes (e.g., salivary changes, oral microbiota, and cytological alterations), and self-reported outcomes (e.g., oral symptoms). Within each domain, results were summarized descriptively, taking into account study characteristics, exposure definitions, and methodological quality.

### 2.8. Risk of Bias Assessment

The methodological quality of the included analytical cross-sectional studies was assessed using the Joanna Briggs Institute (JBI) Critical Appraisal Checklist for Analytical Cross-Sectional Studies [22,28]. Although both cross-sectional and retrospective observational studies were included, the JBI Critical Appraisal Checklist for Analytical Cross-Sectional Studies was applied due to the comparable methodological structure of the included designs. Retrospective observational studies were considered methodologically similar in terms of exposure and outcome assessment based on existing data sources. This approach ensured consistency and comparability across studies in the risk-of-bias assessment.

This tool evaluates eight methodological domains: (1) clarity of inclusion criteria; (2) detailed description of study participants and setting; (3) validity and reliability of exposure measurement; (4) use of objective and standard criteria for outcome assessment; (5) identification of potential confounding factors; (6) strategies to address confounding factors; (7) validity and reliability of outcome measurement; and (8) appropriateness of statistical analysis.

Each domain was rated as “Yes,” “No,” or “Unclear.” For the purpose of overall risk of bias classification, studies were categorized according to the number of domains rated as “No” or “Unclear.” Studies with 0–2 domains rated as “No/Unclear” were considered at low risk of bias, those with 3–4 domains rated as “No/Unclear” were classified as moderate risk of bias, and studies with ≥5 domains rated as “No/Unclear” were considered at high risk of bias. This threshold was established a priori to ensure consistency and transparency in methodological assessment.

Two independent reviewers (C.C.-J. and C.S.-M.) conducted the appraisal of each study. Discrepancies were resolved through discussion and consensus, and when necessary, consultation with a third reviewer.

### 2.9. Analysis of GRADE Evidence Levels

The certainty of evidence was assessed for each evaluated outcome using the GRADE (Grading of Recommendations Assessment, Development and Evaluation) approach [29]. Each outcome was independently evaluated across the following domains: (1) risk of bias, (2) inconsistency, (3) indirectness, (4) imprecision, and (5) publication bias. Three reviewers (C.S.-M., C.M.-P. and C.C.-J.) independently assessed each domain, and any discrepancies were resolved through discussion and consensus.

Due to substantial clinical and methodological heterogeneity across the included studies—including variability in study design, exposure classification (exclusive versus dual use), outcome measurement methods, and reporting of results—a quantitative meta-analysis was not considered appropriate, as it could lead to potentially misleading summary estimates. Therefore, a qualitative synthesis was performed.

## 3. Results

### 3.1. Study Selection

**Figure 1:**
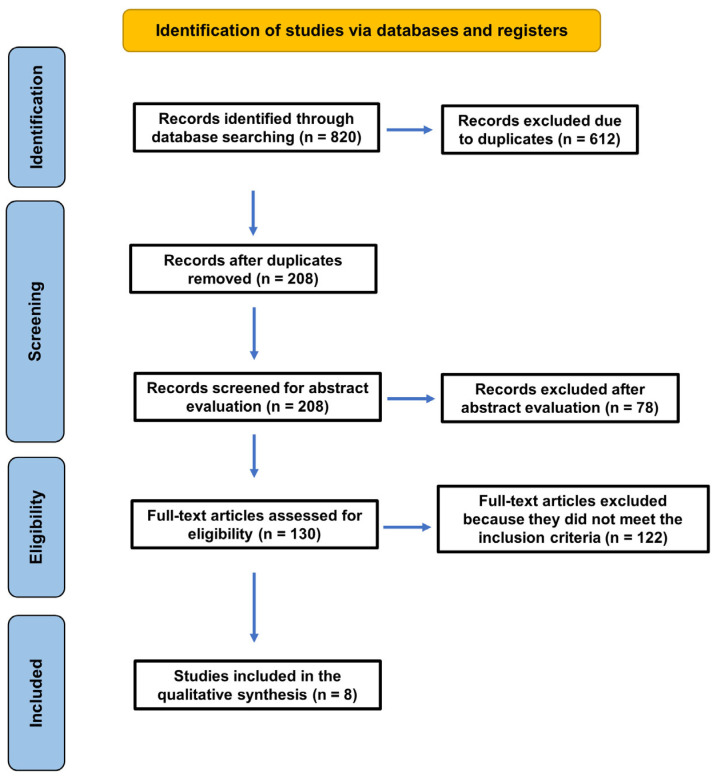
PRISMA 2020 [26] flow diagram illustrating the study selection.

The study selection process is illustrated in Figure 1. The initial database search identified a total of 820 records. A high number of duplicate records was identified due to substantial overlap between the databases searched, which index many of the same journals. After removing duplicate records, 208 remained. After screening titles and abstracts according to the predefined inclusion criteria, 78 items were removed and 130 articles were selected for full-text evaluation. Of these, 122 studies were excluded because they did not meet the inclusion criteria. Finally, 8 studies fulfilled all eligibility criteria and were included in the final qualitative synthesis [30,31,32,33,34,35,36,37]. All included studies were cross-sectional or retrospective observational. No longitudinal or prospective cohort studies meeting inclusion criteria were identified.

### 3.2. Characteristics of the Included Studies

The main characteristics of the included studies are summarized in Table 2. The eight studies were published between 2017 [37] and 2025 [30] and were conducted in different geographical settings, including Europe [36], Asia [31,34,37], Africa [33], Australia [30] and America [32,35]. Sample sizes ranged from 68 participants [36] to 65,528 participants [37]. The largest dataset was based on self-reported data from adolescents, rather than a parent population. Age ranging varied from 12 [37] to 28 [32] years. One included study did not report a precise age range. The proportion of male/female participants was similar in almost all included studies [31,32,33,34,35,36,37].

Exposure classification was heterogeneous across the included studies. Most studies did not clearly distinguish between exclusive e-cigarette users and dual users of combustible tobacco, and exposure was frequently based on self-reported use without detailed characterization of frequency, duration, or prior smoking history. This limitation is summarized in Table 2 and should be considered when interpreting the findings.

#### 3.2.1. Clinical Outcomes

Clinical outcomes assessed across the included studies primarily comprised dental caries, gingival inflammation, and periodontal parameters. Evidence from the included studies suggests that e-cigarette users may present higher levels of dental plaque accumulation and gingival inflammation compared with non-smokers [27,28,31]. Some studies also reported early signs of clinical attachment loss and increased odds of periodontal disease among e-cigarette users [29,31]. However, the magnitude of these findings generally appeared to be lower than that observed in conventional cigarette smokers [29].

Regarding dental caries, several studies indicated a potential association between e-cigarette use and increased caries risk [27,30]. Nevertheless, findings were not entirely consistent across studies, which may be explained by differences in diagnostic criteria, exposure definitions, and study populations.

#### 3.2.2. Biological Outcomes

Biological outcomes included alterations in salivary parameters, oral microbiota composition, and cellular changes in the oral mucosa. Changes in salivary flow and pH were reported in some studies, suggesting a potential impact on the oral environment that may favor demineralization and microbial imbalance [28,30].

In addition, alterations in the oral microbiome were observed, including increased levels of periodontal pathogens such as Porphyromonas gingivalis, indicating a possible shift toward a dysbiotic microbial profile [29,30].

Furthermore, early cellular alterations were identified, including increased frequencies of micronuclei in exfoliated oral epithelial cells, which may reflect subclinical tissue stress or early genotoxic effects associated with e-cigarette exposure [30].

#### 3.2.3. Self-Reported Outcomes

Self-reported outcomes were assessed in several studies through questionnaires [27,28,31]. Commonly reported symptoms among e-cigarette users included xerostomia (dry mouth), oral discomfort, gingival bleeding, and oral ulcers [27,28]. Some participants also reported mucosal changes such as hairy tongue and altered taste perception [28].

However, these findings should be interpreted with caution, as self-reported data may be subject to recall bias and variability in symptom perception [27].

All together, the findings demonstrate considerable heterogeneity across studies in terms of exposure definitions, outcome assessment, and methodological quality, which limits direct comparability and precludes quantitative synthesis.

#### 3.2.4. Confounding Variables

Confounding variables were inconsistently reported across the included studies (Table 3). While some studies accounted for smoking status and, to a lesser extent, oral hygiene practices, other important confounders such as dietary habits, socioeconomic status, and detailed vaping exposure characteristics were rarely assessed or adjusted for. This variability limits the ability to fully interpret the observed associations.

### 3.3. Risk of Bias Assessment

The risk of bias of the included studies was assessed using the Joanna Briggs Institute Critical Appraisal Checklist for Analytical Cross-Sectional Studies. The detailed assessment is presented in Table 4.

Four studies were judged to have a low risk of bias [32,33,35,37], while four studies presented a moderate [30,31,34,36].

The most common source of potential bias was related to insufficient identification or management of confounding factors. Although several studies were classified as having low or moderate risk of bias according to the JBI criteria, important methodological limitations were consistently identified across studies, particularly regarding confounding control, exposure assessment, and reliance on self-reported outcomes. Therefore, the overall methodological quality should be interpreted with caution.

### 3.4. GRADE Assessment of the Certainty of Evidence

The certainty of evidence for each evaluated outcome was assessed using the GRADE (Grading of Recommendations Assessment, Development and Evaluation) approach. The GRADE assessment was conducted at the outcome level, and the certainty of evidence for each outcome is presented in Table 5 with domain-specific justifications. As all included studies were observational in design—predominantly cross-sectional with two retrospective analyses—the certainty of evidence initially started at a low level, in accordance with GRADE guidance for non-randomized studies.

The certainty of evidence was further downgraded due to serious concerns regarding risk of bias. Although half of the included studies were classified as having low overall risk of bias and the remaining as moderate risk according to the JBI criteria, important methodological limitations were consistently present across studies. These included the predominance of cross-sectional designs, inadequate measurement of exposure in some studies, incomplete identification and adjustment for confounding variables, inclusion of dual users of electronic and combustible tobacco products, and reliance on self-reported outcomes in several investigations. Even among studies rated as low risk, residual confounding remained likely, particularly with respect to oral hygiene practices, dietary habits, socioeconomic status, prior dental history, and patterns of tobacco use. Taken together, these limitations were considered sufficiently serious to warrant downgrading the certainty of evidence for risk of bias at the outcome level.

Inconsistency was judged as serious for several outcomes due to heterogeneity in study populations, exposure definitions (exclusive e-cigarette users versus dual users), outcome measurements (clinical examination versus self-reported symptoms), and statistical adjustments. While most studies suggested a directionally similar association between e-cigarette use and poorer oral health indicators, variability in effect sizes and assessment methods limited direct comparability. For outcomes evaluated by a single study (e.g., micronuclei frequency and salivary microbiota alterations), inconsistency could not be formally assessed but remains uncertain due to lack of replication.

Indirectness was considered serious in some outcomes. Several studies included mixed exposure groups in which exclusive e-cigarette users were not clearly differentiated from dual users of electronic and conventional cigarettes. This overlap limits the ability to isolate the independent effect of e-cigarettes on oral health outcomes. Additionally, some studies relied on self-reported oral symptoms rather than standardized clinical examination, which may not fully capture objective oral pathology. This limitation contributed to indirectness and residual confounding in the certainty of evidence assessment.

Imprecision was present in outcomes assessed by small sample sizes (e.g., studies evaluating micronuclei or salivary biomarkers) and in analyses lacking precise effect estimates or confidence intervals. Furthermore, some outcomes were supported by only one or two studies, limiting confidence in the stability of the observed associations.

Although formal assessment of publication bias was not feasible due to the small number of studies per outcome, the possibility of publication bias cannot be excluded, particularly considering the emerging and rapidly evolving nature of e-cigarette research.

Considering the cumulative downgrading for risk of bias, inconsistency, indirectness, and imprecision, the overall certainty of evidence was judged to be low for all evaluated outcomes, including caries risk, periodontal parameters, salivary profile and oral microbiota alterations, oral mucosal changes (micronuclei frequency), and self-reported oral symptoms. This was particularly evident in outcomes supported by a limited number of studies, self-reported measures, or mixed exposure groups. Although the available evidence may suggest a possible association between e-cigarette use and adverse oral health indicators in adolescents and young adults, the predominance of cross-sectional designs, inadequate control of confounding factors, and heterogeneity in outcome assessment preclude causal inference.

Therefore, the findings should be interpreted cautiously, and future well-designed longitudinal studies that clearly differentiate exclusive e-cigarette users from dual users and adequately adjust for relevant confounders are required to strengthen the certainty of evidence in this field.

## 4. Discussion

### 4.1. Main Findings

This systematic review synthesized evidence from eight observational studies evaluating the association between e-cigarette use and oral health outcomes in adolescents and young adults. Overall, the findings may suggest a consistent trend toward poorer oral health indicators among e-cigarette users compared with non-users. Reported alterations included increased caries risk, higher prevalence of gingival inflammation, changes in salivary profile and oral microbiota composition, and early mucosal cellular alterations. However, all included studies were cross-sectional or retrospective in design, and the overall certainty of evidence was low, primarily due to methodological limitations and inadequate control of confounding factors. Therefore, while the observed associations are biologically plausible and directionally consistent, causal inferences cannot be established. Importantly, the uniformly low certainty of evidence does not imply absence of an association, but rather reflects the current methodological limitations of the available studies.

To our knowledge, previous systematic reviews on electronic cigarettes and oral health have predominantly focused on adult populations or have examined isolated oral outcomes [38]. In contrast, the present review specifically addresses adolescents and young adults, a population characterized by ongoing biological development, evolving behavioral patterns, and increasing exposure to flavored and high-nicotine products. This age-specific focus strengthens the relevance of the present findings and highlights important gaps in pediatric and adolescent oral health research.

ECs have become increasingly popular in recent years as an alternative to conventional cigarettes, and data show that the decline in conventional or dual smokers is accompanied by an increase in exclusive EC users [3,4,39]. Although considered safer than traditional cigarettes, ECs have been associated with health risks, including cardiovascular and pulmonary diseases, as well as being a significant risk factor affecting oral health [6].

They are especially popular among young people, attracting adolescents and young adults through exposure to misleading advertising and flavors such as fruits, sweets, and desserts [40]. Moreover, the long-term effects in this population are not fully known.

For clarity, the findings are discussed according to major outcome domains.

#### 4.1.1. Salivary and Microbiological Alterations

The only study included in this review that analyzed salivary profile and oral microbiota in young e-cigarette users was conducted by Kurniawan et al. [30]. This study may indicate alterations in salivary pH, reduced salivary flow rate, and increased levels of Porphyromonas gingivalis. These findings are consistent with previous literature suggesting that salivary acidity is lower in smokers than in non-smokers [41,42]. This phenomenon may be attributed to constant exposure to heated aerosols and nicotine, which may reduce bicarbonate ion secretion [43]. In addition, aldehydes present in aerosols may contribute to pH alterations [44].

The study also suggested that e-cigarette use may reduce salivary flow rates, which is in agreement with previous research [45]. This may be explained by alterations in oral mucosal blood flow and disruption of the salivary reflex. Nicotine may also influence vascularization of salivary glands. Even nicotine-free e-liquids may negatively affect saliva quality due to mucosal irritation [45]. Although differences in salivary viscosity have been reported, the included study did not observe statistically significant changes, although a trend toward higher viscosity was noted [46].

Regarding microbiota, higher levels of Porphyromonas gingivalis were observed among e-cigarette users, consistent with previous findings [47,48,49]. This bacterium thrives in low-oxygen environments, and changes in oral conditions may promote its proliferation [50]. However, these findings should be interpreted cautiously, as they are based on a limited number of studies and may represent indirect evidence rather than definitive mechanistic conclusions.

#### 4.1.2. Mucosal and Cytological Alterations

Evidence regarding mucosal changes was limited to a single study evaluating micronuclei frequency in buccal cells [36]. This study reported higher values among e-cigarette users compared with non-smokers, which may suggest early cellular alterations or epithelial stress. Similar findings have been reported in conventional smokers, where increased micronuclei frequency has been associated with cytotoxic and genotoxic effects [51,52,53]. In addition, increased cellular turnover observed in these contexts may reflect irritation induced by exposure to tobacco-related compounds [54]. However, given the limited number of studies and the young age of participants, these findings should be interpreted as preliminary and hypothesis-generating rather than conclusive.

#### 4.1.3. Periodontal and Clinical Outcomes

With respect to clinical periodontal outcomes, several studies reported higher prevalence of gingival inflammation and early periodontal changes among e-cigarette users [25,26]. These findings are consistent with previous studies indicating increased plaque levels, probing depth, bone loss, inflammatory markers, and gingival crevicular fluid in users of nicotine-containing products [55].

Clinical examinations also identified oral alterations such as nicotine stomatitis and higher prevalence of gingivitis among e-cigarette users [32]. However, variability in clinical assessment methods and incomplete control of confounding factors—such as oral hygiene practices and tobacco use—limit the strength of these observations.

#### 4.1.4. Dental Caries and Hard Tissue Alterations

Evidence from included studies may suggest an increased risk of dental caries and white spot lesions among e-cigarette users [32,35]. This association may be related to the composition of certain e-liquids, which may facilitate bacterial adhesion and biofilm formation [56]. In addition, microbiological shifts toward cariogenic profiles may contribute to demineralization processes.

These findings are consistent with previous reports indicating an increased risk of dental caries among e-cigarette users [57]. However, these interpretations remain speculative and should be considered in light of the observational nature of the evidence and potential confounding factors such as diet and oral hygiene.

#### 4.1.5. Self-Reported Oral Symptoms

Several studies assessed oral health outcomes through self-reported measures, including dry mouth, oral ulcers, taste alterations, and mucosal discomfort [33,34,37]. These studies generally reported a higher prevalence of such symptoms among e-cigarette users.

For example, Alhajj et al. [34] reported a significantly higher prevalence of dry mouth and black tongue among users, while Alade et al. [33] found increased reporting of gingival inflammation, oral ulcers, and xerostomia. Similarly, Cho [37] observed associations between e-cigarette use and oral pain and dental damage.

However, reliance on self-reported data introduces potential recall bias and subjectivity, which may affect the reliability of these findings.

### 4.2. Potential Biological Mechanisms Linking E-Cigarette Use and Oral Health Outcomes

The following section discusses biologically plausible mechanisms that may underlie the observed associations. Taken together, the findings of this systematic review may suggest potential biological pathways through which e-cigarette use could influence oral health in adolescents and young adults [58].

First, alterations in salivary physiology may play an important role. Changes in salivary pH and reduced salivary flow may compromise buffering capacity and mechanical cleansing, thereby creating an environment conducive to demineralization and microbial imbalance. Second, shifts in the oral microbiota, including increased levels of periodontal pathogens such as Porphyromonas gingivalis, may promote a dysbiotic biofilm profile associated with both caries development and periodontal inflammation. Third, inflammatory and early cellular alterations—including increased gingival inflammation and elevated micronuclei frequency in exfoliated oral cells—may indicate that vaping exposure may induce epithelial stress and subclinical tissue damage even in the absence of overt clinical manifestations. These pathways are likely interconnected, with salivary dysfunction facilitating microbial dysbiosis, which in turn may amplify inflammatory responses and early tissue alterations [59].

These proposed mechanisms should be interpreted as biologically plausible hypotheses rather than definitive causal explanations derived from the current evidence.

In addition to these mechanistic considerations, several studies in this review reported self-perceived oral health changes based on questionnaire data. Alhajj et al. [34], in their study conducted among dental students, found that e-cigarette users more frequently reported dry mouth and black hairy tongue compared with non-smokers. Similarly, Alade et al. [33] reported that adolescents and young adults who used e-cigarettes were more likely to experience gingival inflammation, oral ulcers, and xerostomia. The study by Cho [37], which included participants aged 12–18 years, found associations between e-cigarette use and cracked or broken teeth, as well as pain in the tongue or inner cheeks.

These findings may be partially explained by both nicotine-dependent and nicotine-independent mechanisms. Nicotine has been shown to impair mineralization processes in dental tissues and may induce inflammatory responses in human dental pulp cells [51,52]. Independent of nicotine, thermal degradation byproducts from glycerin, propylene glycol, and flavoring agents may contribute to mucosal irritation and discomfort. However, these interpretations should be considered cautiously given the reliance on self-reported outcomes and the observational nature of the evidence [60,61,62,63].

Recent studies have further highlighted the role of e-cigarette use in disrupting oral microbial and metabolic homeostasis. Emerging evidence suggests that vaping may induce functional changes within the subgingival microbiome and alter host–microbe interactions, potentially contributing to gingival inflammation and periodontal imbalance [64,65]. These findings support the hypothesis that e-cigarette exposure may not only modify microbial composition but also influence metabolic activity and inflammatory pathways within the oral environment. However, given the observational nature and heterogeneity of the available studies, these mechanisms should be interpreted as biologically plausible rather than definitively established [66].

### 4.3. Heterogeneity of Exposure and Confounding Factors

An important consideration across all outcome domains is the heterogeneity of exposure definitions and study characteristics. Several included studies involved mixed exposure groups, in which exclusive e-cigarette users were not consistently distinguished from dual users of electronic and combustible tobacco products. This overlap limits the ability to isolate the independent effect of vaping on oral health outcomes and represents one of the main limitations of the current evidence base.

In addition, the inconsistent reporting of key confounding variables—such as smoking status, oral hygiene behaviors, dietary habits, and socioeconomic factors—limited the ability to fully assess their influence on the observed associations. Even studies classified as low risk of bias were subject to residual confounding and limitations inherent to observational designs.

Another important source of heterogeneity relates to the variability of e-cigarette products and patterns of use, which may significantly influence oral health outcomes. Moreover, the heterogeneity of electronic nicotine delivery systems (ENDS), including variability in device types, brands, and e-liquid compositions, represents an additional source of bias. The rapidly evolving and multifactorial nature of these products limits the standardization of exposure assessment and complicates comparisons across studies [3]. Differences between nicotine-containing and nicotine-free e-cigarettes should be considered. While nicotine has been shown to impair vascularization, reduce salivary flow, alter immune response, and interfere with mineralization processes in dental tissues, nicotine-free products are not biologically inert. Thermal degradation of humectants such as propylene glycol and glycerin, as well as flavoring agents, can generate aldehydes and reactive compounds capable of inducing oxidative stress, epithelial irritation, and inflammatory responses in the oral cavity. Therefore, both nicotine-dependent and nicotine-independent mechanisms may contribute to the observed oral alterations. These mechanisms should be interpreted as biologically plausible hypotheses rather than definitive causal explanations derived from the current evidence.

Furthermore, frequency and intensity of vaping are likely important modifiers of risk. Most included studies did not stratify participants according to duration of use, daily puff frequency, or years of exposure. As a result, a potential dose–response relationship cannot be excluded, and heavier or more prolonged use may lead to cumulative salivary, microbiological, and periodontal changes that may not yet be detectable in younger or occasional users.

Finally, device type represents an additional source of variability. Disposable devices, often characterized by higher nicotine salt concentrations and widespread use among adolescents, may deliver greater nicotine exposure per puff compared to certain refillable systems. Conversely, modifiable pod or tank devices allow users to adjust voltage and temperature, potentially increasing aerosol production and thermal degradation byproducts. These differences may influence both chemical exposure and biological responses in oral tissues. Future research should therefore clearly specify product type, nicotine concentration, and usage patterns to better elucidate the independent impact of e-cigarettes on oral health.

### 4.4. Clinical Implications

The findings of this review may have important implications for clinical practice. Oral health professionals may play a key role in identifying early signs of oral alterations associated with e-cigarette use and in providing preventive counseling and cessation support. The potential association between oral lesions and e-cigarette use may also encourage adolescents and young adults to seek dental care, offering an opportunity to detect these conditions and offer appropriate cessation counselling. The findings of this SR indicate that for early detection and intervention.

In this context, dentists may contribute effectively to tobacco and vaping cessation efforts among younger populations. However, widespread misconceptions regarding the safety of e-cigarettes—particularly among adolescents and young adults—may explain their increasing popularity. Evidence suggests that vaping is often perceived as a less harmful or even beneficial alternative for overall health, which may reduce risk awareness and delay behavioral change. This highlights the urgent need for targeted educational strategies and the implementation of prevention and cessation programs addressing the long-term health effects of e-cigarette use [67].

From a public health and clinical perspective, recent guidelines and position statements from pediatric and international health organizations have raised concerns about the increasing use of nicotine products among adolescents and young adults. These reports emphasize that e-cigarettes are not harmless alternatives and may contribute to nicotine dependence and long-term health risks in this population [68]. In addition, recent evidence highlights heterogeneous patterns of nicotine use and dependence among youth, underscoring the complexity of exposure and the need for targeted prevention and cessation strategies [69,70,71].

### 4.5. Limitations

Certain limitations should be noted. First, all included studies were cross-sectional or retrospective in design, which precludes causal inference and necessitates cautious interpretation of the findings. In addition, the use of a single critical appraisal tool across different observational study designs may represent a methodological limitation. Although the Joanna Briggs Institute checklist for analytical cross-sectional studies was applied to ensure consistency in the assessment process, its use for retrospective observational studies may not fully capture design-specific sources of bias. Therefore, this approach may have influenced the precision of the risk-of-bias evaluation and should be considered when interpreting the findings. Even studies classified as low risk of bias were subject to residual confounding and limitations inherent to observational research.

Although the review focused on adolescents and young adults, some variability in age reporting across studies may affect population homogeneity. Additionally, in some studies, the number of daily e-cigarette users was relatively small, which may limit statistical robustness.

Another important limitation relates to the reliance on self-reported outcomes in several studies. Three studies assessed oral health through questionnaires without clinical examination, which may introduce recall bias and subjective interpretation. Although in one study participants were dental students, meaning they could easily identify any oral alterations and whether these changes, if present, could be induced by ECs, it would also have been necessary to evaluate participants’ previous dental history [28], potentially improving the accuracy of self-reported findings. The absence of standardized clinical assessment and information on prior dental history remains a limitation.

Confounding factors were not consistently addressed across studies. Not all included studies accounted for relevant variables such as diet, oral hygiene practices, frequency and duration of vaping, or previous dental conditions. In addition, the inconsistent reporting of key confounding variables—such as smoking status, dual use, dietary habits, and socioeconomic factors—limited the ability to fully assess their influence on the observed associations. The inconsistent reporting and adjustment of key confounding variables—such as smoking status, oral hygiene behaviors, dietary habits, and socioeconomic factors—represent an important limitation of the current evidence base and may have influenced the observed associations.

Finally, an important limitation concerns the inclusion of participants with mixed exposure profiles. Several studies included dual users of e-cigarettes and conventional tobacco products without clear differentiation from exclusive e-cigarette users. This represents a major source of confounding, as combustible tobacco use is a well-established risk factor for oral disease. The inability to separate these exposure groups limits the capacity to isolate the independent effect of e-cigarette use and may lead to overestimation or misinterpretation of observed associations. Furthermore, inadequate adjustment for smoking intensity, duration of use, or previous tobacco exposure further restricts causal interpretation.

### 4.6. Future Directions

Future research should prioritize clearer characterization of exposure groups, with explicit differentiation between exclusive e-cigarette users, dual users, and never-smokers. This distinction is essential to allow more precise estimation of the independent contribution of e-cigarettes to oral pathology, which remains a major limitation of the current evidence base.

Given that most participants included in this review were adolescents and young adults, many potential long-term consequences of e-cigarette use may not yet be fully observable. This highlights the need for well-designed longitudinal studies with extended follow-up periods to better assess the progression and clinical relevance of oral health alterations associated with e-cigarettes.

In addition, future studies should adopt more robust methodological designs, including prospective cohort and case–control approaches, to strengthen causal inference. Oral health outcomes should be assessed through standardized clinical dental examinations, supported by comprehensive anamnesis that includes medical history, dental status, dietary habits, oral hygiene practices, and detailed exposure characteristics such as vaping frequency, duration, and device type.

Furthermore, a better understanding of the behavioral and social determinants underlying e-cigarette and tobacco use among adolescents and young adults is needed. Such insights may contribute to the development of more effective prevention strategies and targeted smoking cessation programs tailored to this population.

## 5. Conclusions

The available evidence suggests a potential association between e-cigarette use and adverse oral health indicators in adolescents and young adults, including alterations in salivary profile, oral microbiota composition, periodontal parameters, and early mucosal changes. However, these findings should be interpreted with caution.

The overall certainty of evidence was low across all evaluated outcomes, primarily due to methodological limitations, including the predominance of cross-sectional designs, inconsistent exposure definitions, inadequate control of confounding factors, and reliance on self-reported measures in several studies. In addition, the frequent inclusion of mixed exposure groups limits the ability to isolate the independent effect of e-cigarette use.

Although the observed associations are biologically plausible and directionally consistent, current evidence is insufficient to establish causal relationships. Therefore, the findings of this review should be considered as indicative rather than conclusive.

Given the increasing prevalence of e-cigarette use among adolescents and young adults, these findings highlight the need for greater clinical awareness and preventive strategies. Future research based on well-designed longitudinal studies with standardized outcome assessment and precise exposure characterization is essential to better understand the impact of e-cigarettes on oral health.

## Figures and Tables

**Table 1:** Complete search strategies for all databases included in the systematic review.

PubMed	(“Electronic Nicotine Delivery Systems”[MeSH] OR “e-cigarette*” OR “electronic cigarette*” OR vaping OR “vape*” OR “ENDS”) AND (“Oral Health”[MeSH] OR “Dental Caries”[MeSH] OR “Periodontal Diseases”[MeSH] OR gingivitis OR periodontitis OR caries OR “oral microbiota” OR saliva OR xerostomia OR “oral lesion*” OR “oral mucosa” OR “micronuclei”) AND (“Adolescent”[MeSH] OR “Young Adult”[MeSH] OR adolescent* OR “young adult*” OR youth OR student*) Filters applied: Humans; English; last 10 years
Scopus	TITLE-ABS-KEY (“e-cigarette*” OR “electronic cigarette*” OR vaping OR “vape*” OR “electronic nicotine delivery systems” OR ENDS) AND TITLE-ABS-KEY (“oral health” OR “dental caries” OR “periodontal disease*” OR gingivitis OR periodontitis OR caries OR “oral microbiota” OR saliva OR xerostomia OR “oral lesion*” OR “oral mucosa” OR micronuclei) AND TITLE-ABS-KEY (adolescent* OR “young adult*” OR youth OR student*) Filters applied: Year (last 10 years); Language: English; Document type: Article
EMBASE	(‘electronic nicotine delivery system’/exp OR ‘electronic cigarette’/exp OR ‘e-cigarette*’:ab,ti OR ‘electronic cigarette*’:ab,ti OR vaping:ab,ti OR ‘vape*’:ab,ti OR ENDS:ab,ti) AND (‘oral health’/exp OR ‘dental caries’/exp OR ‘periodontal disease’/exp OR gingivitis:ab,ti OR periodontitis:ab,ti OR caries:ab,ti OR ‘oral microbiota’:ab,ti OR saliva:ab,ti OR xerostomia:ab,ti OR ‘oral lesion*’:ab,ti OR ‘oral mucosa’:ab,ti OR micronuclei:ab,ti) AND (‘adolescent’/exp OR ‘young adult’/exp OR adolescent*:ab,ti OR ‘young adult*’:ab,ti OR youth:ab,ti OR student*:ab,ti) Filters applied: Humans; English; last 10 years
WoS	TS = (“e-cigarette*” OR “electronic cigarette*” OR vaping OR “vape*” OR “electronic nicotine delivery systems” OR ENDS) AND TS = (“oral health” OR “dental caries” OR “periodontal disease*” OR gingivitis OR periodontitis OR caries OR “oral microbiota” OR saliva OR xerostomia OR “oral lesion*” OR “oral mucosa” OR micronuclei) AND TS = (adolescent* OR “young adult*” OR youth OR student*) Filters applied: Year (last 10 years); Language: English; Document type: Article
Google scholar	Google Scholar was searched using simplified keyword combinations due to platform limitations. The first 200 results sorted by relevance were screened. (“e-cigarette” OR vaping OR “electronic cigarette” OR “electronic nicotine delivery systems”) AND (“oral health” OR caries OR gingivitis OR periodontitis OR saliva OR xerostomia OR “oral microbiota” OR “oral lesions”) AND (adolescent OR “young adult” OR youth OR student)
OpenGrey	(e-cigarette OR vaping OR “electronic cigarette”) AND (“oral health” OR caries OR gingivitis OR periodontitis OR saliva) AND (adolescent OR “young adult” OR youth) OpenGrey was searched using simplified keyword combinations due to platform limitations. All available results were screened manually.

**Table 2:** Characteristics of the included studies.

Authors, Year and Country	Design	Sample Size/Sex (%)	Age Range (y)	Exposure Classification	Oral Aspect Studied	Methods for Studying Oral Status	Results and Conclusions
Kurniawan et al. [30] 2025, Australia	Cross-sectional study	390 p (68% male)	17–25 y	Mixed/unclear separation of exclusive EC and former conventional cigarette users	Saliva Profile and oral microbiota	Collecting saliva samples	EC smokers exhibit higher salivary pH and lower flow rate compared to non-smokers. The analysis of oral bacteria showed higher levels of Porphyromonas gingivalis.
Ibraheem et al. [31] 2024 Saudi Arabia	Cross-sectional study	292 p (54% male)	18–25 y	E-cigarette users; dual use not clearly excluded	Periodontal condition	Getting a clinical exam	Participants showed 64% had gingivitis and 13.3% had periodontitis. The results show more gingival inflammation parameters related to participants who are using EC.
Soares et al. [32] 2024, Brazil	Cross-sectional study	300 p (32% male)	21–28 y	Mixed tobacco/e-cigarette exposure groups	Tooth Loss, caries, WSL, gingivitis, gingival recession, nicotine stomatitis, dentinoenamel staining and coated tongue	Getting a clinical exam	EC use being the most prevalent form, and may lead to detrimental effects on the oral cavity, such as caries and gingivitis.
Alade et al. [33] 2022, Nigeria	Cross-sectional study	2870 p (51% male)	11–23 y	Mixed/self-reported EC use; smoking status not fully differentiated	Gingival inflammation, changes in taste, oral ulcers and dry mouth	Filling out a questionnaire of self-perceived changes	Those who used EC had 1.5 times higher odds of reporting oral lesions. Gingival inflammation, oral ulcers, change in taste, and dry mouth are oral lesions reported by adolescents and young people who use EC.
Alhajj et al. [34] 2022, Yemen	Cross-sectional study	5697 p (40% male)	NR	Mixed/self-reported EC use; dual use not clearly excluded	Sore mouth, dry mouth mouth and/or tongue, inflammation, black tongue, gingivitis	Filling out a questionnaire of self-perceived changes	Reported frequencies of complaints ranged from as low as 3.3% for tongue inflammation with significant differences between EC users and non-users. Compared to non-smokers, EC users reported significantly higher prevalence of dry mouth (33.1% vs. 23.4%) and black tongue (5.9% vs. 2.8%). Dental students showed good oral hygiene practices, but E-cigarette users showed more health complications.
Irusa et al. [35] 2022, USA	Retrospective observational study	846 p (48% male)	16–20 y	E-cigarette/vape users vs. non-users; dual use not clearly excluded	Caries risk level	Getting a clinical exam	There was an association between use of e-cigarettes or vapes and caries risk level of patients; e-cigarettes patients had a higher risk of developing caries.
Pop et al. [36] 2021, Romania	Cross-sectional study	68 p (56% male)	18–24 y	E-cigarette users vs. non-smokers; dual use not clearly specified	Mucosa Alterations	Collecting buccal cells	EC users had significantly higher values of micronuclei and micronucleated cells compared to nonsmokers. The micronuclei count can be used as an early indicator for alterations of oral mucosa and exfoliative cytology represents an accessible tool which could be applied for mass screening.
Cho [37] 2017, Korea	Retrospective observational study	65,528 p (52% male)	12–18 y	Self-reported EC use; dual use not clearly differentiated	gingival pain and/or bleeding, tongue and/or inside-cheek pain, cracked/broken tooth	Filling out a questionnaire of self-perceived changes	When comparing EC users with non-users, there was association for cracked/broken and tongue and/or inside-cheek pain. However, EC use among adolescents was not associated with gingival pain and/or bleeding when adjusted for the potential confounders.

NR: not registred; p: participants; y: years; WSL: white spot lesions; EC: e-cigarettes. Exposure classification was inconsistently reported across studies, and most studies did not clearly distinguish exclusive e-cigarette users from dual users of combustible tobacco.

**Table 3:** Reporting and adjustment of key confounding variables across included studies.

Study	Smoking Status/Dual Use	Oral Hygiene	Diet	Socioeconomic Status	Vaping Exposure	Confounders Adjusted in Analysis
Kurniawan et al. [30] 2025, Australia	Partial	Not reported	Not reported	Not reported	Partial	No
Ibraheem et al. [31] 2024, Saudi Arabia	Not clearly reported	Not reported	Not reported	Not reported	Partial	No
Soares et al. [32] 2024, Brazil	Yes	Not reported	Not reported	Not reported	Partial	Partial
Alade et al. [33] 2022, Nigeria	Partial	Not reported	Not reported	Not reported	Not reported	Partial
Alhajj et al. [34] 2022, Yemen	Partial	Yes	Yes	Yes	Not reported	No
Irusa et al. [35] 2022, USA	Partial	Not reported	Not reported	Not reported	Not reported	Partial
Pop et al. [36] 2021, Romania	Partial	Not reported	Not reported	Not reported	Not reported	No
Cho [37] 2017, Korea	Yes	Not reported	Not reported	Not reported	Not reported	Yes

Yes = clearly reported and/or adjusted; Partial = reported incompletely or considered indirectly; Not reported = no clear information provided in the study or in the extracted data.

**Table 4:** Risk of bias assessment.

Study	Inclusion Criteria Clearly Defined	Participants and Setting Adequately Described	Exposure Measured Validly and Reliably	Objective/Standard Outcome Criteria Used	Confounders Identified	Strategies to Address Confounders	Outcomes Measured Validly and Reliably	Appropriate Statistical Analysis	No/Unclear Domains (n)	Overall Risk of Bias
Kurniawan et al. [30]	Yes	Yes	No	No	No	No	Yes	Yes	4	Moderate
Ibraheem et al. [31]	Yes	Yes	No	No	No	No	Yes	Yes	4	Moderate
Soares et al. [32]	Yes	No	Yes	Yes	No	Yes	Yes	Yes	2	Low
Alade et al. [33]	Yes	Yes	Yes	No	No	Yes	Yes	Yes	2	Low
Alhajj et al. [34]	Yes	Yes	No	No	No	No	Yes	Yes	4	Moderate
Irusa et al. [35]	Yes	Yes	Yes	No	No	Yes	Yes	Yes	2	Low
Pop et al. [36]	Yes	Yes	No	No	No	No	Yes	Yes	4	Moderate
Cho [37]	Yes	Yes	Yes	No	No	Yes	Yes	Yes	2	Low

**Table 5:** GRADE analysis.

Outcome	Relevant Studies	Observed Effect	Quality of Evidence (GRADE)	Comments
Salivary profile and oral microbiota alterations	Kurniawan et al. [30]	Higher salivary pH, reduced salivary flow rate, increased Porphyromonas gingivalis levels among EC users compared to non-users	Low	Started at low (observational design). Downgraded for serious risk of bias (no control of confounders), serious imprecision (single study, moderate sample size), and indirectness (limited geographic population; unclear exclusive vs. dual use). No upgrading criteria met.
Periodontal disease (gingivitis/periodontitis parameters)	Ibraheem et al. [31]; Soares et al. [32]	Higher prevalence of gingival inflammation and early periodontal changes among EC users	Low	Started at low. Downgraded for serious risk of bias (cross-sectional design; incomplete confounder adjustment), inconsistency (heterogeneous periodontal assessment methods), and indirectness (dual users included in some analyses). No evidence of dose–response or large effect size.
Caries risk/White spot lesions	Irusa et al. [35]; Soares et al. [32]	Increased caries risk levels and higher prevalence of white spot lesions among EC users	Low	Started at low. Downgraded for serious risk of bias (observational data; residual confounding by diet and oral hygiene), indirectness (mixed exposure groups), and imprecision (limited number of studies). Clinical assessment strengthens objectivity but does not compensate for design limitations.
Oral mucosal cellular alterations (micronuclei frequency)	Pop et al. [36]	Higher micronuclei and micronucleated cell counts among EC users	Low	Started at low. Downgraded for serious imprecision (single small cross-sectional study), risk of bias (limited confounder control), and indirectness (dental student population limits generalizability). Objective cytological measurement noted but insufficient for upgrading.
Self-reported oral lesions (ulcers, gingival inflammation, taste changes)	Alade et al. [33]	Higher odds of reported oral lesions among EC users	Low	Started at low. Downgraded for serious risk of bias (self-reported outcomes; recall bias), inconsistency (heterogeneous symptom definitions), and indirectness (pandemic context; mixed tobacco exposure).
Self-reported oral symptoms (dry mouth, tongue pain, cracked/broken teeth)	Cho [37]; Alhajj et al. [34]	Greater prevalence of xerostomia, oral discomfort, and cracked/broken teeth among EC users	Low	Started at low. Downgraded for serious risk of bias (self-reported measures; incomplete confounder adjustment), indirectness (dual use not consistently excluded), and imprecision (variability in effect size reporting). Adjustment for confounders reduced some associations in one study.
Overall oral health status and association with other tobacco products	Soares et al. [32]	EC users showed poorer overall oral health indicators compared to non-users	Low	Started at low. Downgraded for indirectness (dual users included; inability to isolate exclusive EC effect), and risk of bias (cross-sectional design; limited adjustment for behavioral confounders). No upgrading criteria fulfilled.

## Data Availability

No new data were created or analyzed in this study.

## Supplementary Materials

The following supporting information can be downloaded at: https://www.mdpi.com/article/10.3390/jcm15103886/s1, Table S1: PRISMA 2020 Checklist [26].
